# External validation of the Scandinavian guidelines for management of minimal, mild and moderate head injuries in children

**DOI:** 10.1186/s12916-018-1166-8

**Published:** 2018-10-12

**Authors:** Johan Undén, Stuart R. Dalziel, Meredith L. Borland, Natalie Phillips, Amit Kochar, Mark D. Lyttle, Silvia Bressan, John A. Cheek, Jocelyn Neutze, Susan Donath, Stephen Hearps, Ed Oakley, Sarah Dalton, Yuri Gilhotra, Franz E. Babl, on behalf of the Paediatric Research in Emergency Departments International Collaborative (PREDICT)

**Affiliations:** 1 Department of Emergency Medicine, Royal Children’s Hospital, 50 Flemington Rd, Parkville, Victoria 3052 Australia; 2 Murdoch Children’s Research Institute, Melbourne, 50 Flemington Rd, Parkville, Victoria 3052 Australia; 3 Department of Paediatrics, Faculty of Medicine, Dentistry and Health Sciences, University of Melbourne, Melbourne, Grattan St, Parkville, Victoria 3010 Australia; 4 Emergency Department, Princess Margaret Hospital for Children, Roberts Rd, Subiaco, Perth, Western Australia 6008 Australia; 5 Divisions of Paediatrics and Emergency Medicine, School of Medicine, University of Western Australia, 35 Stirling Hwy, Crawley, Western Australia 6009 Australia; 6 Emergency Department, Lady Cilento Children’s Hospital, Brisbane and Child Health Research Centre, School of Medicine, The University of Queensland, 501 Stanley St, South Brisbane, Queensland 4101 Australia; 7 Emergency Department, Women’s & Children’s Hospital, Adelaide, 72 King William St, North Adelaide, South Australia 5006 Australia; 8 Emergency Department, The Children’s Hospital at Westmead, 212 Hawkesbury Rd, Westmead, New South Wales 2145 Australia; 9 Emergency Department, Monash Medical Centre, 246 Clayton Rd, Clayton, Victoria 3186 Australia; 10 Emergency Department, Kidzfirst Middlemore Hospital, 100 Hospital Rd, Auckland, 2025 New Zealand; 11 Emergency Department, Starship Children’s Health, 2 Park Rd, Grafton, Auckland, 1023 New Zealand; 12 Liggins Institute, University of Auckland, 85 Park Ave, Grafton, Auckland, 1023 New Zealand; 13 Emergency Department, Bristol Children’s Hospital, Paul O’Gorman Building, Upper Maudlin St, Bristol, BS2 8BJ UK; 14 Academic Department of Emergency Care, University of the West of England, Blackberry Hill, Bristol, BS16 1XS UK; 15 Department of Women’s and Children’s Health, University of Padova, Via Giustiniani3, 2, 35128 Padova, Padova Italy; 16 Department of Operation and Intensive Care, Hallands Hospital, Halmstad, Sweden; 17 Lund University, Lund, Sweden

**Keywords:** Head trauma, Head injury, Guideline, Clinical decision rule, Infant, Child, Computed tomography, Scandinavia

## Abstract

**Background:**

Clinical decision rules (CDRs) aid in the management of children with traumatic brain injury (TBI). Recently, the Scandinavian Neurotrauma Committee (SNC) has published practical, evidence-based guidelines for children with Glasgow Coma Scale (GCS) scores of 9–15. This study aims to validate these guidelines and to compare them with other CDRs.

**Methods:**

A large prospective cohort of children (< 18 years) with TBI of all severities, from ten Australian and New Zealand hospitals, was used to assess the SNC guidelines. Firstly, a validation study was performed according to the inclusion and exclusion criteria of the SNC guideline. Secondly, we compared the accuracy of SNC, CATCH, CHALICE and PECARN CDRs in patients with GCS 13–15 only. Diagnostic accuracy was calculated for outcome measures of need for neurosurgery, clinically important TBI (ciTBI) and brain injury on CT.

**Results:**

The SNC guideline could be applied to 19,007/20,137 of patients (94.4%) in the validation process. The frequency of ciTBI decreased significantly with stratification by decreasing risk according to the SNC guideline. Sensitivities for the detection of neurosurgery, ciTBI and brain injury on CT were 100.0% (95% CI 89.1–100.0; 32/32), 97.8% (94.5–99.4; 179/183) and 95% (95% CI 91.6–97.2; 262/276), respectively, with a CT/admission rate of 42% (mandatory CT rate of 5%, 18% CT or admission and 19% only admission). Four patients with ciTBI were missed; none needed specific intervention. In the homogenous comparison cohort of 18,913 children, the SNC guideline performed similar to the PECARN CDR, when compared with the other CDRs.

**Conclusion:**

The SNC guideline showed a high accuracy in a large external validation cohort and compares well with published CDRs for the management of paediatric TBI.

## Background

Traumatic brain injury (TBI) is a major global health problem [1] with a general incidence of 262 per 100,000 per year [2], which does not seem to be declining despite increased knowledge and prevention strategies [3]. TBI is common in both developed and also in low- and middle-income countries and is associated with considerable mortality and morbidity [3, 4]. The incidence of TBI is higher in children than in adults [5], children are often more difficult to assess and neuroradiological management is associated with concerning health risks [6, 7].

Initial management is focused on the detection or exclusion of significant brain injury, in particular injuries that would need neurosurgical procedures. The gold-standard investigation is computed tomography (CT), which reliably detects and excludes intracranial complications following head injury. However, the concerns of economic, logistic and radiation burden of increasing CT use limits its use for all children with head injury [8–11]. An alternative option is admission to hospital of intermediate risk groups for clinical observation with deferred CT imaging if signs and symptoms worsen or do not improve, a practice which has been demonstrated to be safe but may be associated with higher health care costs [12, 13].

Clinical decision rules (CDRs) have been developed to stratify patients according to the risk of important outcomes and hence indication for CT, with the goal of optimising resource use while assuring detection of important intracranial injuries. Several CDRs for children have been developed including the Pediatric Emergency Care Applied Research Network (PECARN) rule, the Canadian Assessment of Tomography for Childhood Head Injury (CATCH) rule and the Children’s Head Injury Algorithm for the Prediction of Important Clinical Events (CHALICE) rule [14–16]. These were derived using high-quality methods and have recently been externally validated in a large prospective cohort [17]. Although the PECARN rule seems to display the best accuracy [17], in particular a very high sensitivity for relevant outcomes, the actual impact of such a rule will depend on the target population and baseline management routines. Although not borne out by recent data [18, 19], there is an ongoing concern that these rules may increase CT use in some settings [20].

**Fig. 1:**
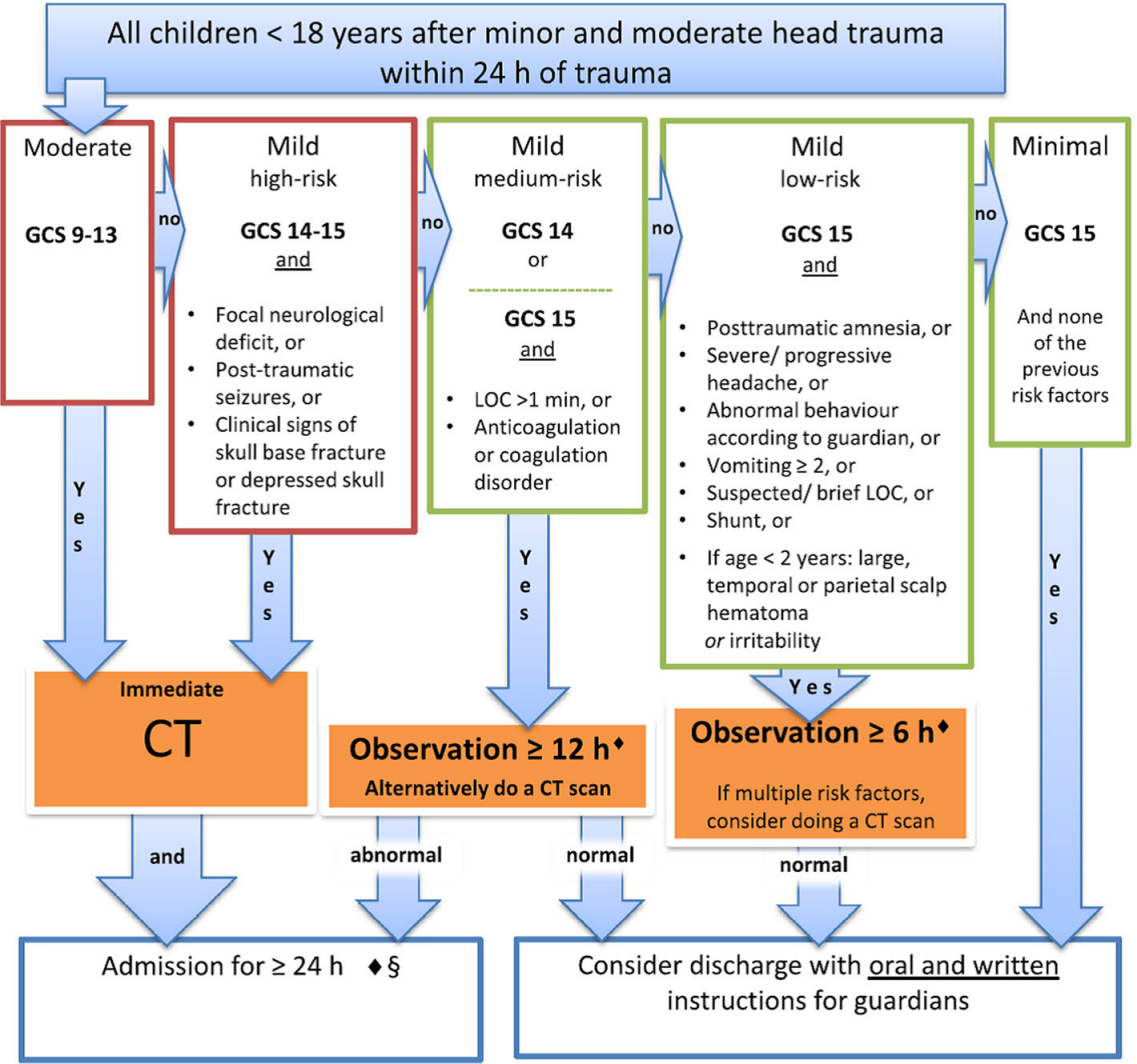
The Scandinavian Neurotrauma Committee (SNC) guideline for management of children (< 18 years) with minimal, mild and moderate traumatic brain injury (TBI) [20]. GCS Glasgow Come Scale, LOC loss of consciousness, CT computed tomography. Modified from Astrand et al. [20]

Recently, the Scandinavian Neurotrauma Committee (SNC), a non-profit organisation of neurosurgeons, anaesthesiologists, intensivists, neurologists and other specialties from Sweden, Norway, Denmark, Finland and Iceland, with an interest in TBI, developed and published evidence-based guidelines for management of minimal, mild and moderate head injuries in adults [21] and children [20]. These guidelines offer a comprehensive guide to TBI management, including selection of patients for CT scan and/or hospital observation, in the context of the Scandinavian health care system, see Fig. 1***.*** As these guidelines were not based on a derivation cohort, validation, in particular external validation, is required before widespread clinical implementation.

Recently, Babl et al. published an appropriately powered multicentre validation and comparison study, the Australian Paediatric Head Injury Rules Study (APHIRST), comparing the accuracy of the PECARN, CHALICE and CATCH CDRs [17, 22]. This study included sufficient predictor variables to externally validate the SNC guidelines. In addition to an external validation as the primary aim, we set out to compare the SNC guidelines to the PECARN, CHALICE and CATCH CDRs.

## Methods

### Design and setting

The APHIRST study was a prospective multicentre observational study, which enrolled 20,137 children (age < 18 years) with head injury of all severities at ten Australian and New Zealand centres of the Paediatric Research in Emergency Departments International Collaborative (PREDICT) network [23]. Predictor variables from the PECARN, CATCH and CHALICE were collected, and the performance accuracy of these rules was externally validated. Detailed information on this study can be found in the primary publication [16] and the protocol publication [22].

The SNC guideline is intended for all children (< 18 years) with head injury and a GCS of 9–15, presenting within 24 h of injury [20]. Being a tool for selecting children for imaging, those children who have already had imaging are excluded.

### Procedure

In most cases, the clinical predictors elicited in the APHIRST study were identical to the ones used in the SNC guideline. In the few instances where variables were different, assumptions were made a priori to analysis, see Table 1. SNC guideline parameters were applied to the APHIRST dataset, and suggested management was noted.

**Table 1:** Comparison of inclusion criteria, exclusion criteria and clinical predictors between the Australasian Paediatric Head Injury Rules Study (APHIRST) cohort and the Scandinavian Neurotrauma Committee (SNC) guidelines

APHIRST	SNC
Inclusion criteria	
All children < 18 years, all GCS	All children < 18 years with head injury within 24 h of trauma, GCS 9–15
Exclusion criteria	
Trivial facial injury only	Prior imaging
Referral from ER to external provider	
Neuroimaging before transfer to site	
Did not wait to be seen	
Predictor variables	
GCS 9–13	GCS 9–13
GCS 14	GCS 14
Positive focal neurology	Focal neurological deficit
Seizure in patient with no history of epilepsy	Post-traumatic seizures
(Clinical signs of basal skull fracture) OR (suspicion of penetrating or depressed skull injury)	Clinical signs of skull base fracture or depressed skull fracture
LOC > 5 s	LOC > 1 min
Any bleeding disorder or anticoagulation therapy	Anticoagulation or coagulation disorder
Amnesia (antegrade or retrograde; > 5 min)	Post-traumatic amnesia
(Severe headache) OR (history of worsening headache)	Severe/progressive headache
Not acting normally per parent report	Abnormal behaviour according to guardian
Vomiting ≥ 2 episodes	Vomiting ≥ 2 episodes
Any or suspected LOC	Suspected/brief LOC
Shunt	Shunt
(Age < 2 and irritability on examination) OR (age < 2 and temporal or parietal hematoma) OR (age < 2 and large, boggy scalp hematoma)	If age < 2 years, large, temporal or parietal scalp hematoma OR irritability
Combination of at least two risk factors from the SNC predictors	Multiple risk factors

*GCS* Glasgow Come Scale, *ER* emergency room, *LOC* loss of consciousness

As with the APHIRST parent publication [17], the SNC guideline was assessed in two ways. Firstly, the cohort was inputted into the SNC guideline according to the guideline inclusion criteria and with the intended SNC primary outcomes, neurosurgical intervention and intracranial injury [20]. Secondly, the same comparison cohort used in the parent publication [17], i.e. children with a GCS of 13–15 who presented within 24 h of injury, was used in order to compare the SNC guideline with PECARN, CHALICE and CATCH CDRs. The common outcome variable used to compare the accuracy of the SNC guideline and the three CDRs was the presence of clinically important TBI (ciTBI) [14].

### Definitions

Neurosurgery was defined as any neurosurgical procedure for TBI.

ciTBI was defined according to the PECARN definition; death from TBI, neurosurgical intervention for TBI, intubation of more than 24 h from TBI or hospital admission of two nights or more for TBI, associated with TBI on CT [14].

TBI on CT was defined as any acute intracranial finding revealed on CT that was attributable to acute injury, including closed depressed skull fractures and pneumocephalus, but excluding non-depressed skull fractures and basilar skull fractures [14].

As the SNC guideline recommends both CT and/or hospital admission with observation, depending on the risk group, a binary variable was assumed where CT and/or observation was compared to discharge. This is similar to the method used for the external validation of the PECARN rule [17].

### Analysis

We did not undertake a separate sample size calculation beyond the sample size calculation undertaken for the APHIRST parent study [22].

Sensitivity, specificity and predictive values were calculated with corresponding 95% confidence intervals. Differences between risk groups were assessed by Fisher’s exact test.

## Results

From the original sample of 20,137 children, we applied SNC guideline eligibility criteria and excluded 1013 children who presented > 24 h after injury and 117 with GCS < 9. Therefore, a total of 19,007 children (94% of the total cohort) were applicable to the SNC guideline. Selected patient characteristics are shown in Table 2.

**Table 2:** Patient characteristics in the entire Australasian Paediatric Head Injury Rules Study (APHIRST) cohort, the APHIRST comparison cohort and the patients eligible for the Scandinavian Neurotrauma Committee (SNC) guideline

	APHIRST validation	APHIRST comparison	SNC
	*n* = 20,137	*n* = 18,913	*n* = 19,007
DEMOGRAPHICS			
Mean age	5.7 (sd 4.7)	5.7 (sd 4.6)	5.7 (sd 4.6)
< 2 years	5374 (26.7%)	5046 (26.7%)	5067 (26.7%)
≥ 2 years	14,763 (73.3%)	13,867 (73.3%)	13,940 (73.3%)
Boys	12,828 (63.7%)	12,073 (63.8%)	12,136 (63.9%)
Girls	7309 (36.3%)	6840 (36.2%)	6871 (36.1%)
Injury mechanism			
Fall	14,119 (70.1%)	13,337 (70.5%)	13,401 (70.5%)
Motor vehicle incident	849 (4.2%)	745 (3.9%)	759 (4.0%)
High-impact projetile or object	1320 (6.6%)	1228 (6.5%)	1232 (6.5%)
Suspected non-accidental injury	112 (0.6%)	81 (0.4%)	85 (0.4%)
High-energy/velocity trauma	1669 (8.3%)	1523 (8.1%)	1543 (8.1%)
Predictor examples			
GCS3–8	121 (0.6%)	–	–
GCS 9–13	231 (1.2%)	132 (0.7%)	226 (1.2%)
GCS 14	578 (2.9%)	567 (3.0%)	567 (3.0%)
GCS 15	19,207 (95.4%)	18,214 (96.3%)	18,214 (95.8%)
LOC	2707 (13.5%)	2468 (13.0%)	2506 (13.2%)
Vomiting	3452 (17.1%)	3094 (16.4%)	3138 (16.5%)
Headache	4127 (20.5%)	3785 (20.0%)	3810 (20.0%)
Multiple risk factors	2597 (12.9%)	2324 (12.3%)	2359 (12.4%)
Outcomes			
Cranial CT	2106 (10.5%)	1691 (8.9%)	1760 (9.3%)
Admission	4544 (22.6%)	4164 (22.0%)	4229 (22.2%)
ER discharge	15,594 (77.4%)	14,749 (78.0%)	14,778 (77.8%)
Neurosurgery	83 (0.4%)	24 (0.1%)	32 (0.2%)
Death	15 (0.1%)	1 (< 0.01%)	1 (< 0.01%)
Clinically important TBI (PECARN)	280 (1.4%)	160 (0.8%)	183 (1.0%)
Clinically significant intracranial injury (CHALICE)	403 (2.0%)	251 (1.3%)	276 (1.5%)

*GCS* Glasgow Come Scale, *ER* emergency room, *LOC* loss of consciousness, *CT* computed tomography, *TBI* traumatic brain injury, *PECARN* Paediatric Emergency Care Applied Research Network, *CHALICE* Children’s Head Injury Algorithm for the Prediction of Important Clinical Events, *NS* neurosurgery, *ciTBI* clinically important traumatic brain injury, *sd* standard deviation

### Validation of SNC guideline

**Fig. 2:**
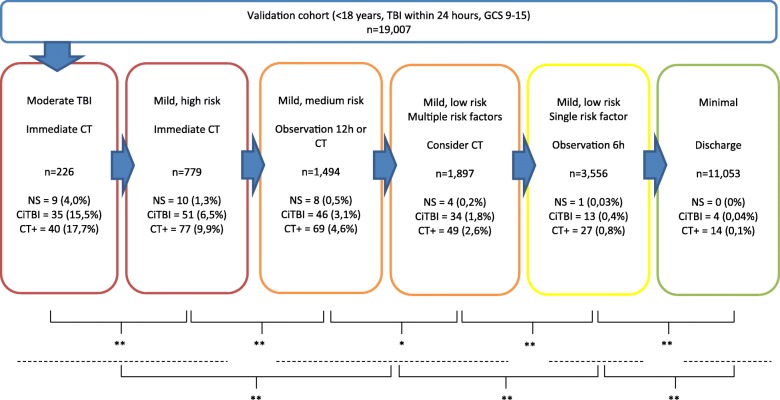
Distribution of children from the validation cohort (*n* = 19,007) in the different Scandinavian Neurotrauma Committee (SNC) guideline risk groups. Corresponding outcomes are provided with percentages. GCS Glasgow Come Scale, CT computed tomography, TBI traumatic brain injury, NS neurosurgery, ciTBI clinically important traumatic brain injury, CT+ brain injury on CT (see text for details). *p < 0.05, **p < 0.001 for differences of ciTBI between groups (Fisher’s exact test)

Thirty-two (0.17%) children needed neurosurgery, 183 (1.0%) had ciTBI, 276 (1.5%) had a TBI on CT and one patient died (TBI was not the cause of death in this patient). The distribution of children in the different SNC risk categories, with corresponding neurosurgery, ciTBI and brain injury on CT outcomes, is shown in Fig. 2. There were significant differences between the risk groups in terms of ciTBI frequency. When combining groups to represent the recommendations of ‘immediate CT’, ‘observation or CT’, ‘observation’ and ‘discharge’, there were also highly significant differences, see Fig. 2. In the primary analysis of the SNC guideline, point sensitivities for the detection of neurosurgery, ciTBI and TBI on CT were 100, 98 and 95%, respectively, and point specificities were 58, 59 and 59%, respectively (Table 3).

**Table 3:** Performance of the Scandinavian Neurotrauma Committee (SNC) guidelines in the validation cohort (*n* = 19,007)

Outcome	Neurosurgery^a^	ciTBI^a^	Brain injury on CT^a^
SNC CT or observation, with outcome	32	179	262
SNC CT or observation, without outcome	7921	7775	7692
SNC discharge, with outcome	0	4	14
SNC discharge, without outcome	11,052	11,049	11,039
Sensitivity (95% CI)	100% (89.1–100)	97.8% (94.5–99.4)	94.9% (91.6–97.2)
Specificity (95% CI)	58.3% (57.5–59.0)	58.7% (58.0–59.4)	58.9% (58.2–59.6)
PPV (95% CI)	0.4% (0.3–0.6)	2.3% (1.9–2.6)	3.3% (2.9–3.7)
NPV (95% CI)	100% (100–100)	100% (99.9–100)	99.9% (99.8–99.9)

*CT* computed tomography, *PPV* positive predictive value, *NPV* negative predictive value, *ciTBI* clinically important traumatic brain injury

^a^See text for detailed definitions

### SNC guideline comparison with CDRs

Of 18,913 children included in the comparison cohort, we further omitted patients with a GCS of 9–12. Twenty-four (0.13%) children needed neurosurgery, 160 (0.85%) had ciTBI, 251 (1.3%) had TBI on CT and one patient died. Point sensitivities and specificities for the detection of neurosurgery, ciTBI and TBI on CT were similar to the validation cohort (Table 4). Four patients with ciTBI and 14 with TBI on CT were missed by the SNC guideline ***(***Table 5). All the missed ciTBIs were classified as such due to admission to hospital > 2 days for TBI, with none needing any specific intervention.

**Table 4:** Performance of the PECARN, CATCH, CHALICE and SNC guidelines in the comparison cohort with all children presenting within 24 h of injury and GCS 13–15 (*n* = 18,913)

	PECARN						CATCH			CHALICE			SNC		
	< 2 years			2 years											
	*n* = 5046			*n* = 13,867											
Primary outcome															
		Positive	Negative		Positive	Negative		Positive	Negative		Positive	Negative		Positive	Negative
Clinically important traumatic brain injury *	Yes	42	0	Yes	117	1	Yes	147	13	Yes	148	12	Yes	156	4
	No	2047	2957	No	6606	7143	No	5560	13,193	No	4018	14,735	No	7704	11,049
Sens (95% CI)	42/42			117/118			147/160			148/160			156/160		
	100∙0% (91∙6–100∙0)			99∙2% (95∙4–100∙0)			91∙9% (86∙5–95∙6)			92∙5% (87∙3–96∙1)			97.5% (93.7–99.3)		
Spec (95% CI)	2957/5004			7143/13749			13,193/18753			14,735/18753			11,049/18753		
	59∙1% (57∙7–60∙5)			52∙0% (51∙1–52∙8)			70∙4% (69∙7–71∙0)			78∙6% (78∙0–79∙2)			58.9% (58.2–59.6)		
PPV (95% CI)	42/2089			117/6723			147/5707			148/4166			156/7860		
	2∙0% (1∙5–2∙7)			1∙7% (1∙4–2∙1)			2∙6% (2∙2–3∙0)			3∙6% (3∙0–4∙2)			2.0% (1.7–2.3)		
NPV (95% CI)	2957/2957			7143/7144			13,193/13206			14,735/14747			11,049/11053		
	100∙0% (99∙9–100∙0)			100∙0% (99∙9–100∙0)			99∙9% (99∙8–99∙9)			99∙9% (99∙9–100∙0)			100% (99∙9–100∙0)		
Secondary outcomes															
		Positive	Negative		Positive	Negative		Positive	Negative		Positive	Negative		Positive	Negative
Traumatic brain injury on CT*	Yes	70	0	Yes	180	1	Yes	220	31	Yes	227	24	Yes	237	14
	No	2019	2957	No	6543	7143	No	5487	13,175	No	3939	14,723	No	7623	11,039
Sens (95% CI)	70/70			180/181			220/251			227/251			237/251		
	100∙0% (94∙9–100∙0)			99∙4% (97∙0–100∙0)			87∙6% (82∙9–91∙5)			90∙4% (86∙1–93∙8)			94.4% (90.8–96.9)		
Spec (95% CI)	2957/4976			7143/13686			13,175/18662			14,723/18662			11,039/18662		
	59∙4% (58∙0–60∙8)			52∙2% (51∙4–53∙0)			70∙6% (69∙9–71∙3)			78∙9% (78∙3–79∙5)			59.2% (58.4–59.9)		
PPV (95% CI)	70/2089			180/6723			220/5707			227/4166			237/7860		
	3∙4% (2∙6–4∙2)			2∙7% (2∙3–3∙1)			3∙9% (3∙4–4∙4)			5∙4% (4∙8–6∙2)			3.0% (2.6–3.4)		
NPV (95% CI)	2957/2957			7143/7144			13,175/13206			14,723/14747			11,039/11053		
	100∙0% (99∙9–100∙0)			100∙0% (99∙9–100∙0)			99∙8% (99∙7–99∙8)			99∙8% (99∙8–99∙9)			99.9% (99.8–99.9)		
		Positive	Negative		Positive	Negative		Positive	Negative		Positive	Negative		Positive	Negative
Neurosurgery*	Yes	6	0	Yes	18 0		Yes	23	1	Yes	22	2	Yes	24	0
	No	2083	2957	No	6705	7144	No	5684	13, 205	No	4144	14,745	No	7835	11,052
Sens (95% CI)	6/6			18/18			23/24			22/24			24/24		
	100∙0% (54∙1–100∙0)			100∙0% (81∙5–100∙0)			95∙8% (78∙9–99∙9)			91∙7% (73∙0–99∙0)			100.0% (85.8–100.0)		
Spec (95% CI)	2957/5040			7144/13849			13,205/18889			14,745/18889			11,052/18889		
	58∙7% (57∙3–60∙0)			51∙6% (50∙7–52∙4)			69∙9% (69∙2–70∙6)			78∙1% (77∙5–78∙6)			58.5% (57.8–59.2)		
PPV (95% CI)	6/2089			18/6723			23/5707			22/4166			24/7859		
	0∙3% (0∙1–0∙6)			0∙3% (0∙2–0∙4)			0∙4% (0∙3–0∙6)			0∙5% (0∙3–0∙8)			0.3% (0.2–0.5)		
NPV (95% CI)	2957/2957			7144/7144			13,205/13206			14,745/14747			11,052/11052		
	100∙0 (99∙9–100∙0)			100∙0% (99∙9–100∙0)			100∙0% (100∙0–100∙0)			100∙0% (100∙0–100∙0)			100∙0% (100∙0–100∙0)		

*PECARN* Paediatric Emergency Care Applied Research Network, *CATCH* Canadian Assessment of Tomography for Childhood Head Injury, *CHALICE* Children’s Head Injury Algorithm for the Prediction of Important Clinical Events, *Sens* sensitivity, *Spec* specificity, *PPV* positive predictive value, *NPV* negative predictive value

^a^See text for detailed definitions

**Table 5:** Characteristics of patients with Glasgow Come Score (GCS) 13–15 presenting within 24 h after injury in the comparison cohort with clinically important traumatic brain injury (CiTBI) not identified by Scandinavian Neurotrauma Committee (SNC) guideline

Age	Gender	GCS	Mechanism of injury	Injury recorded on CT	Neurosurgery	Clinically important traumatic brain injury
6 years	F	15	Fall 1.5 m–3 m	Intracranial haemorrhage/contusion—extra-axial Pneumocephalus Skull fracture—non depressed	No	Yes
						Admitted > 2 days
10 years	F	15	Fall from motorised vehicle	Intracranial haemorrhage/contusion—extra-axial and parenchymal Pneumocephalus Basilar skull fracture	No	Yes
						Admitted > 2 days
15 years	M	15	Unclear	Intracranial haemorrhage/contusion—parenchymal	No	Yes
						Admitted > 2 days
2 years	M	15	Kicked by animal	Intracranial contusion—parenchymal Depressed skull fracture	No	Yes
						Admitted > 2 days

*CT* computed tomography

### CT and observation rate

Applying the SNC guideline would have resulted in a CT/in-hospital observation rate of 42% in both the validation sample and in the comparison cohort. When strictly applied, the mandatory CT rate for the SNC guideline (Fig. 1) would have been only 5% in both the validation and comparison cohorts, with an 18% rate of observation *or* CT and a 19% rate for only observation (no CT). If children with multiple risk factors and medium-risk factors (observation or CT according to the guideline) were all to receive a CT, the rate would be 23%.

## Discussion

In this study, we were able to apply a multinational clinical head injury guideline from Scandinavia to a large, previously collected data set of head injured children and externally assess the accuracy of the guideline. This study appears to adequately validate the accuracy of the SNC guidelines for the management of minimal, mild and moderate head injury in children. In the validation cohort, the guideline displayed a high sensitivity for important outcomes, missing four patients with ciTBI, 14 patients with TBI on CT scan, but no patients requiring neurosurgery out of over 19,000 patients. The SNC guideline was designed to be a pragmatic and universal aid [20]; as demonstrated by the large number of patients, the guideline could be applied to the current APHIRST cohort. Only patients with severe head injury, those who already had neuroimaging and those seeking medical care after 24 h are excluded by the guideline.

When comparing the applicability of the SNC guideline with well-known CDRs, when used as designed [24], the SNC guideline was applicable to a high percentage of the patient cohort (94%); similar to the CHALICE rule (99.5%), a rule including all severities of head injury, and more inclusive than the CATCH (24.6%) and PECARN rules (75.3%) [17]. Adherence to clinical guidelines and CDRs may be problematic [25, 26], especially when dealing with specific and multiple inclusion criteria for guideline applicability [24]. A pragmatic guideline with near-universal inclusion is therefore desirable to ensure clinical use as intended.

Comparing guidelines is difficult due to the differing inclusion criteria, clinical predictors and outcome variables used. Using the APHIRST dataset, a comparison cohort (identical to the SNC inclusion criteria with the exception of patients with GCS 9–12) could be used to directly compare the accuracy of the different rules. The performance of the SNC guideline was similar to the PECARN CDR (high sensitivity, lower specificity) rather than the CATCH and CHALICE CDRs (lower sensitivities but higher specificities). However, the confidence intervals overlap, meaning a statistical difference cannot formally be established. Nonetheless, for the outcome of neurosurgery (the primary outcome variable of the SNC guideline and arguably the most important outcome variable in TBI [17, 20]), the SNC guideline was 100% sensitive, with a relatively high lower 95% confidence interval (85.8%) and a higher overall specificity than PECARN. As an evidence-based guideline, the largest individual evidence contributor for the synthesis of the SNC guideline was derived from the PECARN study, which likely explains the similarities in performance.

The projected CT or admission rate for SNC of 42%, in both the validation and comparison sample, is difficult to compare with the CATCH or CHALICE CDRs. Both dichotomise patients into CT/no CT, without consideration of observation, with projected CT rates for CATCH of 30% (using all predictors) and for the CHALICE rule of 22%, for the comparison cohorts. However, as with the PECARN CDR [14], the SNC guideline has both a CT and in-hospital observation management option, depending on the risk group. The rate for mandatory CT (moderate or high-risk mild TBI according to the guidelines) was only 5%, which increases to 23% if children with medium-risk mild TBI or multiple risk factors (observation or CT according to the guideline) were all to receive a CT.

No patients requiring neurosurgery would be discharged according to the SNC guideline. One patient needing neurosurgery was assigned to the 6-h in-hospital observation group and another 12 patients needing neurosurgery to the in-hospital observation or CT groups. The present study did not include necessary details to examine if the SNC observation routines, mandating a CT scan when a fall in GCS or new/progressive neurological symptoms are observed, would have led to a prompt CT scan for these patients. Children are not to be discharged from hospital until their symptoms (i.e. clinical predictors) have resolved [20]. Overall, this approach may be more expensive than a CT option [13], but removes the logistics and potential radiation risks associated with CT scans.

The corresponding numbers for mandatory CT for the PECARN CDR are not known, though the CT rate would probably be higher due to presence of GCS 14 and altered mental status as predictors for mandatory CT. The presence of these risk factors was a major issue when the SNC workgroup were deciding on the adaptation of an external guideline (PECARN) or synthesising a new, evidence-based guideline. We chose to use the latter strategy, as the group found GCS 14 to be too unreliable as a risk factor to recommend a mandatory CT [27–29] and altered mental status too complicated to use effectively, with potential to lead to unacceptable increases in CT rates in Scandinavia. For this reason, allowing an element of physician judgement in the medium-risk group was chosen.

Unlike other guidelines, the SNC stratifies patients into multiple risk groups for important outcomes. This allows physicians to further understand the potential impact of management in patients. Our analysis confirms the stratification, with higher risk groups showing significantly higher rates of important outcome, such as ciTBI, with gradual reduction of these rates with decreasing risk Fig. 2.

High-energy trauma mechanism is not a strict risk factor in the SNC guidelines. These patients are relatively uncommon in Scandinavia and are managed according to separate clinical trauma protocols. Most receive CT scanning and all of these children are admitted. This risk factor was also judged as complicated to use, having a specific definition and often including assessment of fall height, vehicle speed and number of stairs. In the validation cohort, 1543 patients were involved in high-energy trauma, 65 had brain injury on CT, 50 had ciTBI and 9 needed neurosurgery. All patients needing neurosurgery were identified by other predictors included in the SNC guideline. This suggests that omitting this risk factor is safe in the presence of other risk factors included in the guideline.

Children with suspicion of non-accidental injury (NAI) are always admitted to hospital in Scandinavia and generally receive diagnostic imaging. However, this is not a defined risk factor in the actual guideline, although it is clearly stated that these children should be admitted independent of TBI predictors [20].

In adult TBI management, biomarkers, specifically S100B, have been recommended in clinical guidelines as they could reduce CT rates and overall costs [30] and studies in children have shown promise [31]. Such a biomarker would be most valuable in the intermediate risk groups, such as the medium- and low-risk groups from the SNC guidelines (i.e. the groups presently managed with in-hospital observation), especially considering that today’s clinical predictors seem to have reached their full potential for decision making. Indeed, the actual CT rate from the APHIRST study was only 8.9% in the comparison cohort [17], indicating that clinical guidelines may have limited effect in situations with high-baseline clinician accuracy and low CT rates [32]. However, the evidence base for S100B is too weak for a clinical recommendation in children. Other potential biomarkers have shown promise in adults [33], but studies in children are lacking.

Ultimately, the choice of a guideline will be dependent on the baseline situation and intended effect in the health care setting. Before the SNC guideline, most Swedish hospitals did not have official management pathways for paediatric head injury [34] and many used the SNC guideline from 2000 [13], intended for adults. Although the PECARN rules are based upon a rigorously powered cohort and are externally validated, Scandinavian experts were reluctant to recommend these rules for clinical practice, instead opting for a pragmatic, universal and comprehensive evidence-based option [20]. The results of this study support this approach.

The main strength of this study is the large dataset which was robustly powered and prospectively collected. Also, the dataset was adopted into the guideline by an author (SH) unconnected with the SNC group. However, a limitation is that the dataset was not designed with the SNC guidelines in mind (published first after the study was commenced), but to assess the accuracy of PECARN, CHALICE and CATCH. Clinical predictors were, however, identical in most cases. The few cases where clinical predictors were approximated would likely not have affected the overall performance of the guideline. Additionally, the clinical setting was that of Australian and New Zealand emergency predominately tertiary departments, which may differ from care in the Scandinavian countries for which the SNC guideline was developed.

## Conclusion

In this study, we were able to apply the clinical SNC head injury guideline to a large, previously collected data set of head injured children. The evidence-based SNC head injury guideline was externally assessed in terms of its accuracy and found to have a high sensitivity, missing very few patients with ciTBI and none needing neurosurgery. The present validation study supports the clinical use of the guideline, although national validation in Scandinavian countries may also be warranted.

## Abbreviations

CATCH: Canadian Assessment of Tomography for Childhood Head Injury
CDR: Clinical Decision Rule
CHALICE: Children’s Head Injury Algorithm for the Prediction of Important Clinical Events
ciTBI: Clinically important TBI
CT: Computed tomography
PECARN: Pediatric Emergency Care Applied Research Network
PREDICT: Paediatric Research in Emergency Departments International Collaborative
SNC: Scandinavian Neurotrauma Committee
TBI: Traumatic brain injury
